# Integrating Time‐Adjusted Imaging Instability Into Functional Outcome Prediction After Intracerebral Hemorrhage: Development and Validation of the HAGIV Score

**DOI:** 10.1002/acn3.70457

**Published:** 2026-06-25

**Authors:** Lei Song, Ren Ke, Anqi Chen, Yufei Fu, Hang Zhou, Rujia Wang, Liwei Zou, Yongqiang Yu, Ming Yuan, Xiaoming Qiu, Mengzhou Xue

**Affiliations:** ^1^ Department of Cerebrovascular Diseases The Second Affiliated Hospital of Zhengzhou University Zhengzhou Henan China; ^2^ Department of Radiology Huangshi Central Hospital, Affiliated Hospital of Hubei Polytechnic University Huangshi Hubei China; ^3^ Huangshi Key Laboratory of Cerebrovascular Disease Imaging and Artificial Intelligence, Huangshi Central Hospital, Affiliated Hospital of Hubei Polytechnic University Huangshi Hubei China; ^4^ Department of Radiology Xiangyang Central Hospital, Affiliated Hospital of Hubei University of Arts and Science Xiangyang Hubei China; ^5^ Department of Radiology Xiangyang No. 1 People's Hospital, Hubei University of Medicine Xiangyang Hubei China; ^6^ Department of Radiology Tangshan Gongren Hospital Tangshan Hebei China; ^7^ Department of Radiology The Second Affiliated Hospital of Anhui Medical University Hefei Anhui China; ^8^ Department of Radiology The First Affiliated Hospital of Anhui Medical University Hefei Anhui China; ^9^ Office of Medical Research Huangshi Central Hospital, Affiliated Hospital of Hubei Polytechnic University Huangshi Hubei China

**Keywords:** computed tomography, HAGIV score, intracerebral hemorrhage, prognosis, stroke

## Abstract

**Objective:**

Early risk stratification may support clinical decision‐making in spontaneous intracerebral hemorrhage (ICH). We aimed to develop and internally validate HAGIV, a score integrating frequency of imaging markers (FIM), a time‐adjusted non‐contrast computed tomography (CT) metric of hematoma expansion, with established predictors for 90‐day functional outcome in supratentorial ICH.

**Methods:**

This prespecified prognostic modeling study used a multicenter retrospective cohort of consecutive supratentorial ICH patients with baseline non‐contrast CT within 6 h of onset (January 2018–August 2022). The HAGIV score was constructed by assigning integer points according to regression coefficients from multivariable logistic regression. Discrimination for poor functional outcome (modified Rankin Scale score 3–6) was assessed using area under the curve (AUC) and compared with established ICH prognostic scores.

**Results:**

HAGIV incorporated baseline hematoma volume (H), age (A), Glasgow Coma Scale score (G), frequency of imaging markers (I), and presence of intraventricular hemorrhage (V). In the derivation cohort, HAGIV achieved an AUC of 0.86, significantly outperforming the ICH (0.81), MICH (0.81), Outcome (0.72), and Landseed ICH (0.79) scores (all *p* < 0.001, DeLong's test). This superiority was confirmed in the validation cohort, where HAGIV maintained an AUC of 0.84 compared with 0.76, 0.78, 0.75, and 0.75, respectively.

**Interpretation:**

In this predominantly small‐to‐moderate, supratentorial ICH cohort, HAGIV integrated FIM with established prognostic variables and improved discrimination for 90‐day outcome. It may support interpretable early risk stratification for counseling and trial design, but prospective external validation is required before broader clinical implementation.

## 1 Introduction

Spontaneous intracerebral hemorrhage (ICH) remains a devastating cerebrovascular event with high mortality and morbidity. Early risk stratification may support structured management, clinical trial selection, and communication with patients and families [1]. Although several prediction models exist, such as the widely used ICH score, they primarily rely on static indices such as hematoma volume and Glasgow Coma Scale (GCS) score and therefore do not explicitly capture the dynamic pathophysiology of hematoma expansion, a well‐recognized and potentially modifiable determinant of early deterioration and long‐term disability [2, 3, 4, 5, 6].

Accumulating evidence indicates that specific density and shape features on non‐contrast computed tomography (CT) are predictors of hematoma expansion and poor outcome [7]. However, the clinical utility of individual radiological signs is constrained by limited sensitivity, despite their ease of use [8]. To address this limitation, the frequency of imaging markers (FIM) was previously developed as a time‐adjusted metric that normalizes the number of baseline non‐contrast CT markers of active bleeding to the onset‐to‐CT interval [9]. FIM has shown greater predictive sensitivity for hematoma expansion and unfavorable functional outcome than individual CT markers alone [9, 10]. We therefore hypothesized that adding FIM to conventional prognostic variables would improve outcome prediction after ICH. Accordingly, the HAGIV score was developed as an FIM‐integrated prognostic tool that builds on existing ICH scoring frameworks. We developed and internally validated HAGIV for predicting 90‐day functional outcome and compared its performance with established ICH prognostic scales.

## 2 Methods

### 2.1 Study Design, Setting, and Participants

This study was a prespecified prognostic modeling analysis using an existing multicenter retrospective cohort [9, 10]. Patients were recruited between January 2018 and August 2022 from six hospitals across three Chinese provinces: Hubei (Huangshi Central Hospital, Xiangyang No. 1 People's Hospital, and Xiangyang Central Hospital), Anhui (The First and Second Affiliated Hospitals of Anhui Medical University), and Hebei (Tangshan Gongren Hospital). The parent FIM cohort was designed to investigate non‐contrast CT markers of hematoma expansion, which have been primarily characterized and validated in supratentorial hematomas; therefore, the present secondary prognostic modeling analysis was restricted to supratentorial ICH. Eligible patients were adults with spontaneous supratentorial ICH, known symptom‐onset time, baseline non‐contrast CT within 6 h of symptom onset, follow‐up CT within 48 h, and available 90‐day modified Rankin Scale (mRS) assessment. Patients were excluded if they had infratentorial hemorrhage, primary intraventricular hemorrhage, multiple hematomas, secondary ICH due to trauma, aneurysm, vascular malformation, tumor, hemorrhagic transformation, or other structural causes, prior anticoagulant therapy, admission laboratory evidence of coagulopathy, surgical intervention before follow‐up CT, unclear onset time, delayed or missing follow‐up CT, substantial imaging artifacts, or missing 90‐day outcome data. Eligible individuals were randomly assigned in a 7:3 ratio to derivation and validation cohorts using computer‐generated random numbers. The patient‐selection flowchart is provided as Figure S1.

Patient management was determined by treating physicians at each participating center according to local institutional protocols and contemporary ICH guideline recommendations available during the study period, including blood pressure management, neurosurgical evaluation, supportive care, and prevention or treatment of complications; adherence to individual guideline elements was not centrally adjudicated. Because this was a secondary analysis of an existing cohort, detailed information on early care limitation‐including do‐not‐attempt‐resuscitation/do‐not‐resuscitate orders, withdrawal of life‐sustaining treatment, withholding of otherwise indicated intensive interventions, and transition to comfort‐focused care–was not prospectively recorded across the six participating centers. We did not reconstruct care‐limitation or treatment‐intensity variables from incomplete records because their availability, definitions, and documentation practices varied across centers, which could introduce substantial information bias. Consequently, early care‐limitation status could not be reliably ascertained or included as a covariate or stratification variable, and the number of patients with early care limitation could not be reliably reported from this dataset. The study was approved by the institutional or ethics committee of each collaborating center (approval numbers: XYYYE20220081, PJ2022‐09‐30, 2022–22, YX2023‐134, 2021–036, and GRYY‐LL‐KJ2022‐K820). Informed consent was waived because the analysis used de‐identified retrospective data.

### 2.2 Data Collection and Variable Definitions

Prespecified admission variables were grouped into clinical, laboratory, and imaging domains. Clinical data included demographics (age and sex), vascular risk factors and medical history (alcohol use, smoking, diabetes mellitus, hypertension, prior ICH, and prior ischemic stroke), vital signs (systolic and diastolic blood pressures), and neurological status assessed by the GCS score. Laboratory values included platelet count, international normalized ratio, and glucose.

Imaging variables included hematoma location (deep vs. lobar), parenchymal hematoma and intraventricular hemorrhage (IVH) volumes, presence of IVH at baseline, and three CT markers of hematoma instability. Imaging markers were defined as follows: hypodensities, defined as fully enclosed intralesional hypoattenuating foci [11]; blend sign, defined as a clearly demarcated hypodense–hyperdense interface with a density difference ≥ 18 Hounsfield units [12], and island sign, defined as ≥ 3 separate or ≥ 4 connectable perihematomal satellite foci [13]. Two trained readers, an experienced radiologist and a stroke neurologist with 10 and 5 years of experience, respectively, independently reviewed axial CT images while blinded to clinical data and 90‐day outcomes. Disagreements were adjudicated by a senior neuroradiologist with 20 years of experience. As previously reported for this cohort, inter‐reader agreement was substantial to almost perfect for hypodensities, blend sign, and island sign, with Cohen kappa values of 0.84, 0.85, and 0.79, respectively [14]. FIM was calculated as the number of these imaging markers (hypodensities, blend sign, and island sign) divided by onset‐to‐first CT time (hours), serving as a time‐adjusted index of early imaging instability [9]. All CT images were acquired with 5‐mm slice thickness. Hematoma and IVH volumes were measured using semi‐automated volumetric segmentation (3D Slicer, version 4.11.2), with discrepancies resolved by consensus according to our prior workflow [14].

### 2.3 Outcome Definitions

The primary outcome was poor functional outcome at 90 days, defined as mRS score 3–6, representing functional dependence or death. This threshold was prespecified and is commonly used in ICH outcome studies, although dichotomization of mRS outcomes is inherently context dependent. Secondary outcomes were functional dependence or death using a stricter threshold of mRS 4–6 and all‐cause mortality, defined as mRS 6 [1, 15].

### 2.4 Statistical Analysis

Continuous variables are presented as mean ± standard deviations or median (interquartile range) and were compared using the Student's *t*‐test or Mann–Whitney *U* test, as appropriate. Categorical variables are presented as counts (percentages) and were compared using the chi‐square test or Fisher exact test. In the derivation cohort, candidate predictors were selected from baseline clinical, laboratory, and imaging variables. After univariate selection (*p* < 0.10) of factors associated with 90‐day poor outcome (mRS 3–6), multivariable logistic regression was performed to obtain odds ratios (ORs) and 95% confidence intervals (CIs). To facilitate bedside use, continuous predictors were categorized using receiver operating characteristic‐informed thresholds in the derivation cohort and rounded to clinically interpretable cut points. Integer points were assigned proportional to the regression coefficients, and the HAGIV score was defined as the sum of component points. Discrimination was assessed using area under the curve (AUC) with sensitivity, specificity, positive predictive value (PPV), and negative predictive value (NPV). Calibration was assessed by comparing predicted and observed probabilities using calibration plots and was quantified using the Brier score, calibration intercept, calibration slope, calibration error indices, and Spiegelhalter's test in both cohorts. To examine calibration across the full range of functional outcomes, we compared the observed and model‐predicted proportions of patients in each 90‐day mRS category from 0 to 6 in both cohorts. Absolute discrepancies were calculated as the difference between predicted and observed percentages for each mRS category. AUCs were compared between HAGIV and existing ICH prognostic scores (ICH score [2], MICH score [3], Outcome score [4], and Landseed ICH score [5]) using DeLong's test. In addition, to assess whether model performance differed across hematoma severity, we performed a subgroup analysis stratified by baseline hematoma volume using the 30‐mL threshold from the original ICH score. Discrimination and diagnostic performance of HAGIV were evaluated separately in patients with hematoma volume ≤ 30 mL and > 30 mL. All analyses were performed using R (version 4.0.3). Two‐sided *p* < 0.05 was considered statistically significant.

## 3 Results

### 3.1 Study Population and Characteristics

Of 1253 supratentorial ICH patients, 877 and 376 were assigned to the derivation and validation cohorts, respectively; baseline characteristics were well balanced (Table S1). Overall, 56.90% (713/1253) of patients had 90‐day poor outcome (mRS score 3–6), including 58.27% (511/877) in the derivation cohort and 53.72% (202/376) in the validation cohort. The incidences of mRS score 4–6 and mortality were 48.12% and 11.49%, respectively, with no between‐cohort differences. In univariate analyses within the derivation cohort, patients with poor outcome were older, more likely to have hypertension, had lower platelet counts, lower admission GCS scores, higher systolic blood pressure, shorter onset‐to‐first CT time, larger hematoma and IVH volumes, higher frequency of baseline IVH, higher prevalence of hypodensities, blend sign, and island sign, and higher FIM (all *p* < 0.05; Table 1). Collectively, these findings indicated that both baseline clinical severity and early CT‐based imaging instability were strongly associated with unfavorable functional outcomes.

**TABLE 1:** Univariate analysis comparing patients with and without poor outcome in the derivation cohort.

Variables	mRS score 0–2 (*n* = 366)	mRS score 3–6 (*n* = 511)	*p*
Age, Mean ± SD, y	58.67 ± 11.24	62.89 ± 12.31	< 0.001
Sex, male, *n* (%)	238 (65.03)	323 (63.21)	0.580
History of alcohol, *n* (%)	101 (27.60)	133 (26.03)	0.605
History of smoking, *n* (%)	100 (27.32)	140 (27.40)	0.980
History of diabetes mellitus, *n* (%)	38 (10.38)	49 (9.59)	0.698
History of hypertension, *n* (%)	277 (75.68)	346 (67.71)	0.010
History of ICH, *n* (%)	38 (10.38)	56 (10.96)	0.786
History of ischemic stroke, *n* (%)	43 (11.75)	75 (14.68)	0.210
Blood glucose values, Mean ± SD, mmol/L	7.46 ± 3.29	7.76 ± 2.87	0.155
Platelet count, Mean ± SD, 10^9^/L	208.58 ± 58.18	194.43 ± 65.37	< 0.001
International normalized ratio, Mean ± SD	0.96 ± 0.10	0.97 ± 0.11	0.252
GCS score, Median (IQR)	14.00 (12.00–15.00)	10.00 (8.00–12.50)	< 0.001
Systolic blood pressure, Mean ± SD, mm Hg	166.54 ± 24.64	170.74 ± 28.81	0.021
Diastolic blood pressure, Mean ± SD, mm Hg	97.88 ± 16.25	97.30 ± 17.69	0.622
Onset‐to‐first CT time, Median (IQR), h	2.40 (1.58–3.72)	2.15 (1.31–3.27)	0.005
Baseline hematoma volume, Median (IQR), mL	7.94 (4.38–14.00)	17.58 (9.76–30.89)	< 0.001
Baseline IVH volume, Median (IQR), mL	0.00 (0.00–0.00)	0.00 (0.00–3.33)	< 0.001
Hematoma sites, lobar, *n* (%)	65 (17.76)	104 (20.35)	0.337
Presence of IVH at baseline, *n* (%)	59 (16.12)	226 (44.23)	< 0.001
Hypodensities, *n* (%)	78 (21.31)	274 (53.62)	< 0.001
Blend sign, *n* (%)	70 (19.13)	141 (27.59)	0.004
Island sign, *n* (%)	38 (10.38)	183 (35.81)	< 0.001
Frequency of imaging markers	0.00 (0.00–0.41)	0.52 (0.00–1.02)	< 0.001

Abbreviations: CT, computed tomography; GCS, Glasgow Coma Scale; ICH, intracerebral hemorrhage; IQR, interquartile range; IVH, intraventricular hemorrhage; mRS, modified Rankin Scale; SD, standard deviation.

### 3.2 Independent Predictors and Derivation of the HAGIV Score

In multivariable logistic regression, independent factors associated with poor outcome (mRS score 3–6) included age (OR 1.04, 95% CI 1.02–1.06), platelet count (OR 0.99, 95% CI 0.99–0.99), GCS score (OR 0.73, 95% CI 0.68–0.79), baseline hematoma volume (OR 1.08, 95% CI 1.06–1.10), baseline IVH volume (OR 1.11, 95% CI 1.03–1.19), baseline IVH presence (OR 3.67, 95% CI 2.17–6.20), and FIM (OR 3.63, 95% CI 2.45–5.38) (Table 2).

**TABLE 2:** Multivariate analysis for factors associated with poor outcome (mRS score 3–6) in the derivation cohort.

Variables	β	S.E	Z	OR (95% CI)	*p*
Intercept	−0.43	1.04	−0.41	0.65 (0.08–5.01)	0.679
Age, y	0.04	0.01	4.77	1.04 (1.02–1.06)	< 0.001
History of hypertension					
No				1.00 (Reference)	
Yes	−0.25	0.21	−1.20	0.78 (0.52–1.17)	0.232
Platelet count, 10^9^/L	−0.01	0.00	−2.48	0.99 (0.99–0.99)	0.013
GCS score	−0.31	0.04	−8.25	0.73 (0.68–0.79)	< 0.001
Baseline hematoma volume, mL	0.08	0.01	7.25	1.08 (1.06–1.10)	< 0.001
Baseline IVH volume, mL	0.11	0.04	2.88	1.11 (1.03–1.19)	0.004
Presence of IVH at baseline					
No				1.00 (Reference)	
Yes	1.30	0.27	4.85	3.67 (2.17–6.20)	< 0.001
Frequency of imaging markers	1.29	0.20	6.41	3.63 (2.45–5.38)	< 0.001

Abbreviations: CI, confidence interval; GCS indicates Glasgow Coma Scale; IVH, intraventricular hemorrhage; OR, odds ratio; SE, standard error; Z, *z*‐statistic; β, beta coefficient.

For bedside usability, continuous predictors were dichotomized in the derivation cohort using receiver operating characteristic‐derived cutoffs (Table S2): age > 68.5 years, baseline hematoma volume > 12.4 mL, baseline IVH volume > 0.22 mL, GCS ≤ 12.5, platelet count ≤ 178.5 × 10^9^/L, and FIM > 0.27. To reduce collinearity and improve clinical practicality, baseline IVH presence (β = 1.30) was retained over baseline IVH volume (β = 0.11) due to a stronger association and easier applicability. Platelet count was also excluded due to a minimal β coefficient (0.01) and limited clinical relevance.

The HAGIV score was constructed by assigning integer point values proportional to the regression coefficients of the five independent predictors, resulting in a total score ranging from 0 to 11. The score comprises five components: H (baseline hematoma volume), A (age), G (GCS score), I (FIM), and V (baseline IVH present). Points were allocated as follows: age > 68 years (1 point), GCS ≤ 12 (2 points), baseline hematoma volume > 12 mL (2 points), baseline IVH present (3 points), and FIM > 0.27 (3 points). The scoring scheme is detailed in Table 3.

**TABLE 3:** Determinants of the HAGIV score.

Component	Categories	Points
Age, y	≤ 68	0
	> 68	1
GCS score	> 12	0
	≤ 12	2
Baseline hematoma volume, mL	≤ 12	0
	> 12	2
Frequency of imaging markers	≤ 0.27	0
	> 0.27	3
Presence of IVH at baseline	No	0
	Yes	3
Total points		0–11

*Note:* The HAGIV score consists of age, Glasgow Coma Scale score, baseline hematoma volume, frequency of imaging markers, and presence of IVH at baseline.

Abbreviation: IVH, intraventricular hemorrhage.

### 3.3 Risk Stratification and Diagnostic Performance

**FIGURE 1:**
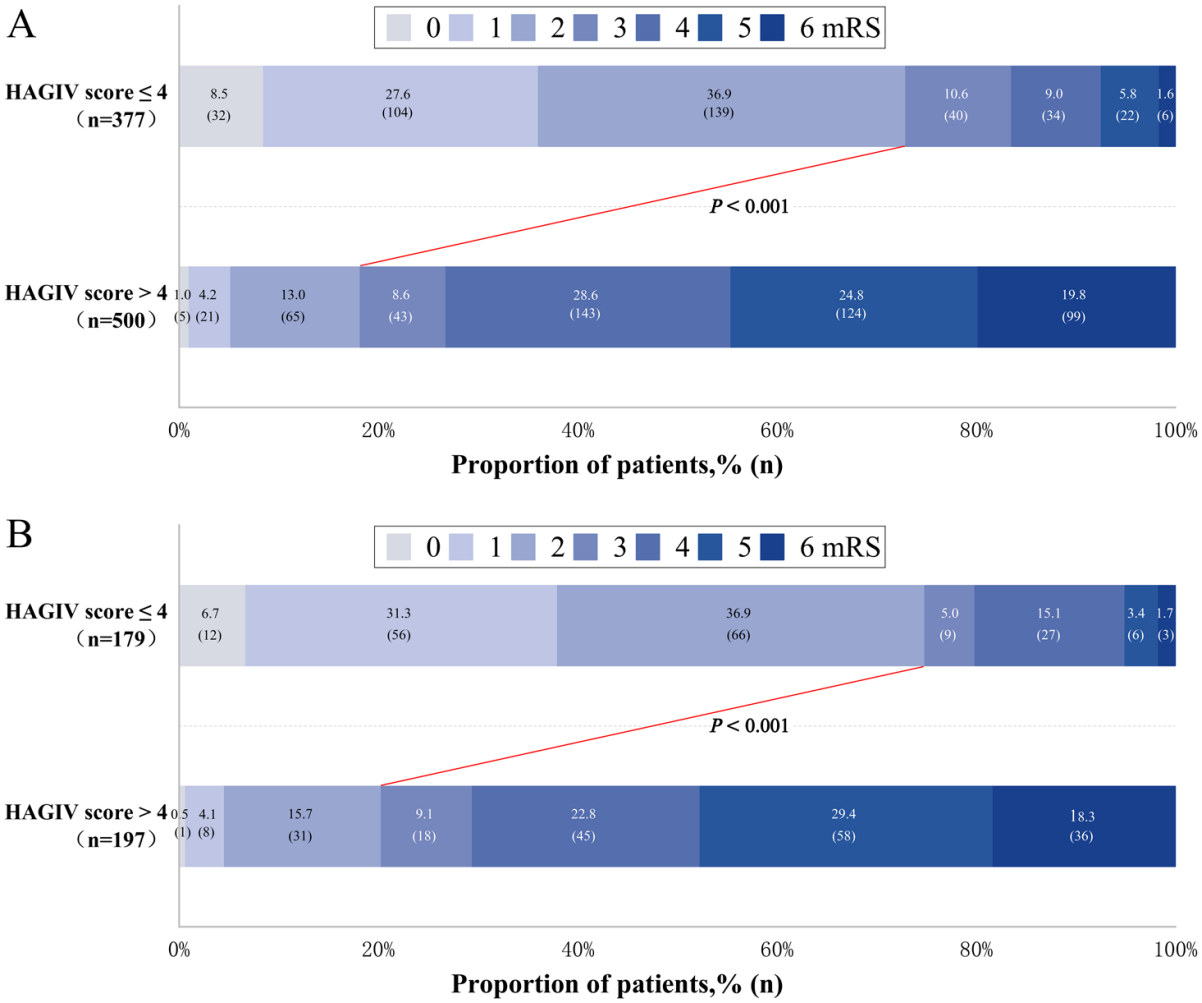
Distribution of modified Rankin Scale (mRS) scores at 90 days after intracerebral hemorrhage, stratified by the HAGIV score (≤ 4 vs. > 4) in the derivation (A) and validation (B) cohorts. The HAGIV score consists of age, Glasgow Coma Scale, baseline hematoma volume, frequency of imaging markers, and presence of intraventricular hemorrhage at baseline. Stacked bars show the percentage of patients in each mRS category, with patient counts provided in parentheses. The solid red line marks the boundary between favorable (mRS 0–2) and poor (mRS 3–6) outcomes.

The proportion of patients with poor outcome (mRS 3–6) increased stepwise with higher HAGIV scores, ranging from 7.07% (score 0) to 100% (scores 9 and 11) in the derivation cohort and from 13.73% (score 0) to 100% (score 10) in the validation cohort. Using the dichotomized threshold (0–4 vs. 5–11), patients with HAGIV 0–4 had substantially lower rates of poor outcome than those with HAGIV 5–11 (derivation: 27.06% vs. 81.80%, Figure 1A; validation: 25.14% vs. 79.70%, Figure 1B). At a threshold of ≥ 5, sensitivity, specificity, PPV, and NPV were 0.80, 0.75, 0.82, and 0.73, respectively, in the derivation cohort, and 0.78, 0.77, 0.80, and 0.75 in the validation cohort. The HAGIV score showed strong discrimination for poor prognosis, with AUCs of 0.86 (95% CI 0.84–0.89) and 0.84 (95% CI 0.80–0.88) in the derivation and validation cohorts, respectively (Table 4).

**TABLE 4:** Proportion of patients experiencing poor outcome (mRS score 3–6) stratified by the HAGIV score.

Variables	Derivation Cohort (*n* = 877)	Validation Cohort (*n* = 376)
Score		
0	7/99 (7.07%)	7/51 (13.73%)
1	2/31 (6.45%)	3/24 (12.50%)
2	12/56 (21.43%)	8/27 (29.63%)
3	46/128 (35.94%)	15/58 (25.86%)
4	35/63 (55.56%)	12/19 (63.16%)
5	103/162 (63.58%)	33/55 (60.00%)
6	58/70 (82.86%)	28/32 (87.50%)
7	93/109 (85.32%)	43/50 (86.00%)
8	67/69 (97.10%)	23/28 (82.14%)
9	10/10 (100.00%)	7/8 (87.50%)
10	43/45 (95.56%)	14/14 (100.00%)
11	35/35 (100.00%)	9/10 (90.00%)
Dichotomized score		
0–4	102/377 (27.06%)	45/179 (25.14%)
5–11	409/500 (81.80%)	157/197 (79.70%)
Dichotomized test characteristics (95% CI)		
Sensitivity	0.80 (0.76–0.83)	0.78 (0.71–0.83)
Specificity	0.75 (0.70–0.79)	0.77 (0.70–0.83)
PPV	0.82 (0.78–0.85)	0.80 (0.73–0.85)
NPV	0.73 (0.68–0.77)	0.75 (0.68–0.81)
AUC	0.86 (0.84–0.89)	0.84 (0.80–0.88)

*Note:* The HAGIV score consists of age, Glasgow Coma Scale score, baseline hematoma volume, frequency of imaging markers, and presence of intraventricular hemorrhage at baseline.

Abbreviations: AUC, area under the curve; CI, confidence interval; NPV, negative predictive value; PPV, positive predictive value.

Calibration analysis showed favorable agreement between predicted and observed risks. The HAGIV score demonstrated excellent calibration in the derivation cohort and generally acceptable calibration in the validation cohort, with no significant evidence of miscalibration by Spiegelhalter's test. Detailed calibration metrics and plots are shown in Table S3 and Figure S2. Figure S3 shows the observed and predicted 90‐day mRS distributions in the derivation and validation cohorts. The predicted proportions closely matched the observed proportions overall. The largest discrepancy was observed in the validation cohort at mRS score 3, where the predicted proportion was 2.61% higher than the observed proportion, followed by mRS score 5, where it was 1.98% lower than the observed proportion.

### 3.4 Comparison With Established ICH Prognostic Scores

**FIGURE 2:**
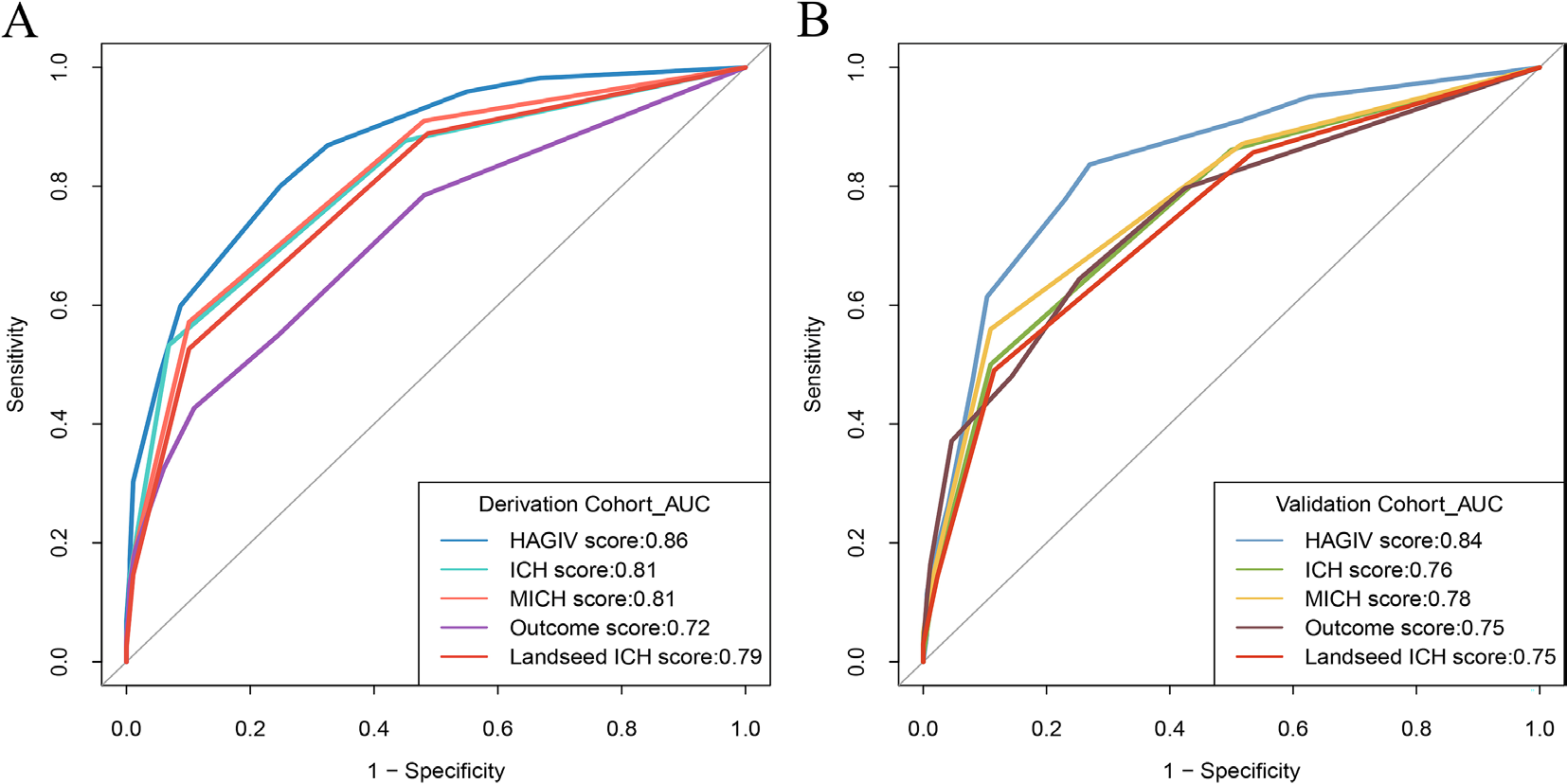
Receiver operating characteristic curves comparing the discriminative performance of the HAGIV score with established prognostic scales for predicting poor 90‐day functional outcome (modified Rankin Scale 3–6) after intracerebral hemorrhage in the derivation (A) and validation (B) cohorts. The HAGIV score consists of age, Glasgow Coma Scale, baseline hematoma volume, frequency of imaging markers, and presence of intraventricular hemorrhage at baseline. The ICH score consists of age, Glasgow Coma Scale, baseline hematoma volume, and hematoma location. The MICH score consists of Glasgow Coma Scale, baseline hematoma volume, and presence of intraventricular hemorrhage at baseline. The Outcome score consists of age, Glasgow Coma Scale, and systolic blood pressure. The Landseed ICH score consists of Glasgow Coma Scale, baseline hematoma volume, and diabetes mellitus.

In the derivation cohort, HAGIV demonstrated superior discrimination for poor outcome (mRS 3–6), achieving an AUC of 0.86 versus 0.81 for the ICH score, 0.81 for the MICH score, 0.72 for the Outcome score, and 0.79 for the Landseed ICH score (all *p* < 0.001, DeLong's test). Similar differences were observed in the validation cohort, where HAGIV maintained an AUC of 0.84 compared with 0.76, 0.78, 0.75, and 0.75, respectively (Table 5 and Figure 2). For secondary clinical endpoints, HAGIV also showed robust discrimination for more severe disability (mRS 4–6) in both cohorts (derivation AUC 0.86, validation AUC 0.83; Table S4). In mortality prediction, HAGIV performed comparably to the ICH, MICH, and Outcome scores and significantly better than the Landseed ICH score in both cohorts (Table S5).

**TABLE 5:** Comparison of predictive power of the HAGIV score and other ICH scores for predicting poor outcome (mRS score 3–6).

ICH scores	Sensitivity (95% CI)	Specificity (95% CI)	PPV (95% CI)	NPV (95% CI)	AUC (95% CI)	DeLong's test
**Derivation Cohort (*n* = 877)**						
HAGIV score	0.80 (0.76–0.83)	0.75 (0.70–0.79)	0.82 (0.78–0.85)	0.73 (0.68–0.77)	0.86 (0.84–0.89)	
ICH score	0.53 (0.49–0.58)	0.93 (0.90–0.96)	0.92 (0.88–0.94)	0.59 (0.55–0.63)	0.81 (0.78–0.83)	< 0.001
MICH score	0.57 (0.53–0.61)	0.90 (0.86–0.93)	0.89 (0.85–0.92)	0.60 (0.56–0.64)	0.81 (0.79–0.84)	< 0.001
Outcome score	0.43 (0.38–0.47)	0.89 (0.85–0.92)	0.84 (0.79–0.89)	0.53 (0.49–0.57)	0.72 (0.69–0.75)	< 0.001
Landseed ICH score	0.53 (0.48–0.57)	0.90 (0.86–0.93)	0.88 (0.84–0.91)	0.58 (0.53–0.62)	0.79 (0.76–0.82)	< 0.001
**Validation Cohort (*n* = 376)**						
HAGIV score	0.78 (0.71–0.83)	0.77 (0.70–0.83)	0.80 (0.73–0.85)	0.75 (0.68–0.81)	0.84 (0.80–0.88)	
ICH score	0.50 (0.43–0.57)	0.89 (0.83–0.93)	0.84 (0.76–0.90)	0.61 (0.54–0.67)	0.76 (0.72–0.81)	< 0.001
MICH score	0.56 (0.49–0.63)	0.89 (0.83–0.93)	0.86 (0.78–0.91)	0.64 (0.57–0.70)	0.78 (0.73–0.82)	0.002
Outcome score	0.48 (0.41–0.55)	0.86 (0.80–0.90)	0.80 (0.71–0.86)	0.59 (0.52–0.65)	0.75 (0.71–0.80)	0.001
Landseed ICH score	0.49 (0.42–0.56)	0.89 (0.83–0.93)	0.83 (0.75–0.89)	0.60 (0.54–0.66)	0.75 (0.70–0.79)	< 0.001

*Note:* The HAGIV score consists of age, Glasgow Coma Scale score, baseline hematoma volume, frequency of imaging markers, and presence of intraventricular hemorrhage at baseline. The ICH score consists of age, Glasgow Coma Scale score, baseline hematoma volume, and hematoma location. The MICH score consists of Glasgow Coma Scale score, baseline hematoma volume, and presence of intraventricular hemorrhage at baseline. The Outcome score consists of age, Glasgow Coma Scale score, and systolic blood pressure. The Landseed ICH score consists of Glasgow Coma Scale score, baseline hematoma volume, and diabetes mellitus.

Abbreviations: AUC, area under the curve; CI, confidence interval; ICH, intracerebral hemorrhage; NPV, negative predictive value; PPV, positive predictive value.

In subgroup analyses stratified by baseline hematoma volume, most patients had hematoma volumes ≤ 30 mL, including 730/877 patients in the derivation cohort and 313/376 patients in the validation cohort. HAGIV maintained good discrimination in the ≤ 30 mL subgroup, with AUCs of 0.84 in the derivation cohort and 0.82 in the validation cohort. Among patients with hematoma volume > 30 mL, HAGIV showed an AUC of 0.86 in the derivation cohort but 0.62 in the validation cohort. Using the prespecified cutoff of HAGIV ≥ 5, sensitivity remained high in the > 30 mL subgroup, whereas specificity was low, reflecting the high prevalence of poor outcome among patients with larger hematomas. Detailed subgroup results are shown in Table S6.

## 4 Discussion

In this multicenter cohort of patients with supratentorial ICH imaged within 6 h of onset, we developed and internally validated the HAGIV score to predict 90‐day functional outcome. HAGIV demonstrated strong discrimination in both derivation and validation cohorts and outperformed commonly used ICH prognostic scales for predicting poor outcome (mRS 3–6).

A core innovation of the HAGIV score is the integration of FIM, a time‐adjusted non‐contrast CT metric intended to reflect hyperacute hematoma instability. Traditional ICH prognostic scales largely quantify baseline injury burden (e.g., age, GCS, hematoma volume, IVH) but do not explicitly encode early hemorrhagic activity [2, 3, 4, 5]. In contrast, FIM is designed to capture early hemorrhage instability by jointly considering marker burden and the time window in which these phenotypes are observed [9]. The independent association between FIM and outcome in multivariable analysis supports the hypothesis that early, time‐conditioned imaging instability provides incremental prognostic information beyond conventional severity indicators. Translating FIM into a bedside score (HAGIV) therefore bridges a mechanistically informed imaging signal with practical clinical decision‐making.

HAGIV incorporates five readily available and clinically intuitive variables–age, GCS, baseline hematoma volume, baseline IVH presence, and FIM–each reflecting distinct dimensions of ICH pathophysiology. Age captures host vulnerability and recovery reserve [16]. Admission GCS reflects neurological injury severity and early mass effect [2]. Baseline hematoma volume quantifies primary parenchymal injury and is consistently one of the strongest predictors across ICH outcome models [2, 3, 4, 5, 17]. Baseline IVH indicates ventricular involvement and secondary injury pathways such as hydrocephalus, elevated intracranial pressure, and neuroinflammation [18]. FIM complements these variables by representing early hemorrhagic instability as expressed on baseline non‐contrast CT [9]. Together, these domains–host factors, clinical severity, hematoma burden, ventricular extension, and dynamic imaging instability–form a coherent and parsimonious framework for outcome prediction that is easily applicable at first clinical contact.

When benchmarked against commonly used prognostic scores (ICH score [2], MICH score [3], Outcome score [4], and Landseed ICH score [5]), HAGIV consistently demonstrated higher discrimination for predicting 90‐day poor functional outcome in both cohorts, and calibration analysis showed good agreement between predicted and observed risks. This improvement should be interpreted as incremental rather than definitive. The original ICH score was developed primarily to stratify ICH severity and facilitate communication, not to serve as a deterministic mortality or functional prognosticator [19]. Moreover, the apparent prognostic performance of ICH scores may be influenced by early care‐limitation decisions in derivation cohorts, introducing a potential self‐fulfilling prophecy effect [20, 21, 22]. Although a modest improvement in discrimination may be clinically useful within structured management frameworks such as CODE ICH and the Integrated ICH Pathway, which emphasize time‐sensitive bundled care, standardized protocols, time‐based performance metrics, and multidisciplinary coordination [23, 24], HAGIV should be interpreted as an admission‐based risk‐stratification tool rather than as a standalone predictor of individual outcome or determinant of treatment decisions. The present cohort was composed predominantly of patients with small‐to‐moderate hematoma volumes. In subgroup analysis using the 30‐mL threshold, HAGIV maintained good discrimination in patients with hematoma volume ≤ 30 mL, but performance was less stable in those with hematoma volume > 30 mL, particularly in the validation cohort. Its utility in large or massive hematomas therefore requires further validation.

HAGIV may have practical value as an admission‐based risk‐stratification tool in neurocritical care. Because it uses variables available at baseline evaluation, it may support structured communication about baseline risk, early multidisciplinary planning, closer monitoring, and trial enrichment. However, HAGIV should be viewed as a decision‐support tool, not as evidence of treatment futility. Early care limitation can directly influence mortality and 90‐day functional status after ICH, and poor outcomes predicted by an admission‐based severity score may partly reflect subsequent treatment intensity rather than disease severity alone. This creates a potential self‐fulfilling prophecy if high predicted risk is used to justify do‐not‐attempt‐resuscitation/do‐not‐resuscitate orders, withdrawal of life‐sustaining treatment, withholding of otherwise indicated intensive interventions, or transition to comfort‐focused care [20, 21, 22]. Consistent with the 2022 AHA/ASA guideline for spontaneous ICH [1], HAGIV, like any baseline prognostic score, should not be used as the sole basis for forecasting individual prognosis or making goals‐of‐care decisions. It should be used only as an adjunct to serial neurological assessment, multidisciplinary clinical judgment, patient values, and shared decision‐making.

Several limitations should be acknowledged. First, although this study included multiple centers and used split‐sample internal validation, its retrospective design may have introduced selection bias, information bias, and residual confounding; prospective external validation in independent healthcare settings is required before broad clinical implementation. Second, HAGIV is an admission‐based risk‐stratification score and does not incorporate post‐admission management, complications, rehabilitation access, discharge disposition, social determinants, or early care‐limitation decisions. Because treatment intensity and care‐limitation variables were not systematically captured across centers, they could not be reliably compared between outcome groups or adjusted for, which may have affected the observed association between admission severity and 90‐day mRS. Third, the cohort consisted predominantly of patients with small‐to‐moderate hematoma volumes, and model performance in patients with large or massive hemorrhages remains uncertain. Fourth, the mRS threshold used to define poor outcome is partly subjective and may vary across populations; although mRS score 3–6 was prespecified and secondary outcomes of mRS score 4–6 and mortality were evaluated, alternative thresholds may be appropriate in other settings. Fifth, 90‐day mRS may not fully capture long‐term recovery after ICH, as meaningful functional improvement can continue beyond 90 days and extend to 1 year or longer in some survivors [25]. Sixth, HAGIV depends on identification of non‐contrast CT markers and hematoma/IVH quantification, so variability in reader expertise and imaging workflows may affect real‐world generalizability. Finally, the cohort was restricted to supratentorial ICH imaged within 6 h of onset. Because infratentorial ICH differs in anatomy, hematoma expansion patterns, clinical course, and prognosis, HAGIV should not be extrapolated to infratentorial hemorrhage or late‐presenting ICH without dedicated validation. Future prospective studies should systematically collect treatment intensity and care‐limitation data, include longer‐term follow‐up, and determine whether HAGIV‐guided care can improve patient‐centered outcomes.

## 5 Conclusions

The HAGIV score showed good internal discrimination and calibration as an admission‐based risk‐stratification tool in patients with supratentorial ICH, particularly in this cohort predominantly composed of small‐to‐moderate hematoma volumes. It may help refine early risk stratification and support trial enrichment, but it should be used only as an adjunct to comprehensive clinical assessment and shared decision‐making. Prospective external validation with systematic collection of treatment intensity, care‐limitation data, and longer‐term outcomes is warranted before routine clinical implementation.

## Author Contributions

Lei Song contributed to writing the original draft. Mengzhou Xue, Xiaoming Qiu, and Ming Yuan take final responsibility for this article. Yufei Fu, Hang Zhou, Rujia Wang, and Liwei Zou contributed to data collection. Ren Ke contributed to imaging editing. Anqi Chen contributed to data analysis. Yongqiang Yu revised the manuscript for important intellectual content.

## Funding

The authors deeply acknowledge the financial support. This work received funding from the National Natural Science Foundation of China (No. U25A2065), Hubei Provincial Natural Science Foundation of China (No. 2024AFB431, No. 2024AFD004, and 2023AFD017), and the Health Commission of Hubei Province Scientific Research Project (No. WJ2025Q071).

## Conflicts of Interest

The authors declare no conflicts of interest.

## Supporting information


**Figure S1:** Study inclusion and exclusion flowchart.
Abbreviations: CT, computed tomography; ICH, intracerebral hemorrhage; and mRS, modified Rankin Scale.
**Figure S2:** Calibration plots of the HAGIV score for predicting poor outcome (mRS score 3–6) in the derivation (A) and validation (B) cohorts.
Abbreviations: Emax, maximum absolute calibration error; E90, 90th percentile absolute calibration error; Eavg, average absolute calibration error; mRS, modified Rankin Scale; HAGIV, hematoma volume, age, Glasgow Coma Scale score, frequency of imaging markers, and intraventricular hemorrhage score.
**Figure S3:** Observed and model‐predicted 90‐day modified Rankin Scale distributions in the derivation (A) and validation (B) cohorts.
**Table S1:** Baseline characteristics of the study.
Abbreviations: CT, computed tomography; GCS, Glasgow Coma Scale; ICH, intracerebral hemorrhage; IQR, interquartile range; IVH, intraventricular hemorrhage; mRS, modified Rankin Scale; and SD, standard deviation.
**Table S2:** Cut‐off values and predictive power for poor outcome (mRS score 3–6) of each continuous variable in the derivation cohort.
Abbreviations: AUC, area under the curve; CI, confidence interval; GCS, Glasgow Coma Scale; IVH, intraventricular hemorrhage; NPV, negative predictive value; PPV, positive predictive value.
**Table S3:** Calibration performance of the HAGIV score for predicting poor outcome (mRS score 3–6) in the derivation and validation cohorts.
Abbreviations: Emax, maximum absolute calibration error; E90, 90th percentile absolute calibration error; Eavg, average absolute calibration error.
**Table S4:** Comparison of predictive power of the HAGIV score and other ICH scores for predicting poor outcome (mRS score 4–6).
Abbreviations: AUC, area under the curve; CI, confidence interval; NPV, negative predictive value; ICH, intracerebral hemorrhage; and PPV, positive predictive value.
The HAGIV score consists of age, Glasgow Coma Scale score, baseline hematoma volume, frequency of imaging markers, and presence of intraventricular hemorrhage at baseline.
The ICH score consists of age, Glasgow Coma Scale score, baseline hematoma volume, and hematoma location.
The MICH score consists of Glasgow Coma Scale score, baseline hematoma volume, and presence of intraventricular hemorrhage at baseline.
The Outcome score consists of age, Glasgow Coma Scale score, and systolic blood pressure.
The Landseed ICH score consists of Glasgow Coma Scale score, baseline hematoma volume, and diabetes mellitus.
**Table S5:** Comparison of predictive power of the HAGIV score and other ICH scores for predicting poor outcome (mortality, mRS score 6).
Abbreviations: AUC, area under the curve; CI, confidence interval; NPV, negative predictive value; ICH, intracerebral hemorrhage; and PPV, positive predictive value.
The HAGIV score consists of age, Glasgow Coma Scale score, baseline hematoma volume, frequency of imaging markers, and presence of intraventricular hemorrhage at baseline.
The ICH score consists of age, Glasgow Coma Scale score, baseline hematoma volume, and hematoma location.
The MICH score consists of Glasgow Coma Scale score, baseline hematoma volume, and presence of intraventricular hemorrhage at baseline.
The Outcome score consists of age, Glasgow Coma Scale score, and systolic blood pressure.
The Landseed ICH score consists of Glasgow Coma Scale score, baseline hematoma volume, and diabetes mellitus.
**Table S6:** Predictive performance of the HAGIV score for poor outcome (mRS Score 3–6) stratified by baseline hematoma volume.
Abbreviations: AUC, area under the curve; CI, confidence interval; NPV, negative predictive value; and PPV, positive predictive value.
The HAGIV score consists of age, Glasgow Coma Scale score, baseline hematoma volume, frequency of imaging markers, and presence of intraventricular hemorrhage at baseline.

## Data Availability

The data that support the findings of this study are available from the corresponding author upon reasonable request.
