# Supplementary material for: Integrating Time‐Adjusted Imaging Instability Into Functional Outcome Prediction After Intracerebral Hemorrhage: Development and Validation of the HAGIV Score
Source: Ann Clin Transl Neurol. 2026 Jun 25:10.1002/acn3.70457. Online ahead of print. doi: 10.1002/acn3.70457 (PMC13394952; doi:10.1002/acn3.70457)
Supplement: Supplementary file 1 — Figure S1: Study inclusion and exclusion flowchart. Abbreviations: CT, computed tomography; ICH, intracerebral hemorrhage; and mRS, modified Rankin Scale. Figure S2: Calibration plots of the HAGIV score for predicting poor outcome (mRS score 3–6) in the derivation (A) and validation (B) cohorts. Abbreviations: Emax, maximum absolute calibration error; E90, 90th percentile absolute calibration error; Eavg, average absolute calibration error; mRS, modified Rankin Scale; HAGIV, hematoma volume, age, Glasgow Coma Scale score, frequency of imaging markers, and intraventricular hemorrhage score. Figure S3: Observed and model‐predicted 90‐day modified Rankin Scale distributions in the derivation (A) and validation (B) cohorts. Table S1: Baseline characteristics of the study. Abbreviations: CT, computed tomography; GCS, Glasgow Coma Scale; ICH, intracerebral hemorrhage; IQR, interquartile range; IVH, intraventricular hemorrhage; mRS, modified Rankin Scale; and SD, standard deviation. Table S2: Cut‐off values and predictive power for poor outcome (mRS score 3–6) of each continuous variable in the derivation cohort. Abbreviations: AUC, area under the curve; CI, confidence interval; GCS, Glasgow Coma Scale; IVH, intraventricular hemorrhage; NPV, negative predictive value; PPV, positive predictive value. Table S3: Calibration performance of the HAGIV score for predicting poor outcome (mRS score 3–6) in the derivation and validation cohorts. Abbreviations: Emax, maximum absolute calibration error; E90, 90th percentile absolute calibration error; Eavg, average absolute calibration error. Table S4: Comparison of predictive power of the HAGIV score and other ICH scores for predicting poor outcome (mRS score 4–6). Abbreviations: AUC, area under the curve; CI, confidence interval; NPV, negative predictive value; ICH, intracerebral hemorrhage; and PPV, positive predictive value. The HAGIV score consists of age, Glasgow Coma Scale score, baseline hematoma volume, frequency of imaging m [file ACN3-9999-0-s001.docx]

**Supplementary Material**

| **Table S1.** Baseline characteristics of the study. | | | | |
| --- | --- | --- | --- | --- |
| **Variables** | **Total**  **(*n* = 1253)** | **Derivation Cohort**  **(*n* = 877)** | **Validation Cohort**  **(*n* = 376)** | ***P*-value** |
| Age, Mean ± SD, y | 61.16 ± 12.28 | 61.13 ± 12.05 | 61.22 ± 12.82 | 0.912 |
| Sex, male, n (%) | 806 (64.33) | 561 (63.97) | 245 (65.16) | 0.687 |
| History of alcohol, n (%) | 330 (26.34) | 234 (26.68) | 96 (25.53) | 0.672 |
| History of smoking, n (%) | 332 (26.50) | 240 (27.37) | 92 (24.47) | 0.287 |
| History of diabetes mellitus, n (%) | 132 (10.53) | 87 (9.92) | 45 (11.97) | 0.279 |
| History of hypertension, n (%) | 877 (69.99) | 623 (71.04) | 254 (67.55) | 0.217 |
| History of ICH, n (%) | 134 (10.69) | 94 (10.72) | 40 (10.64) | 0.966 |
| History of ischemic stroke, n (%) | 160 (12.77) | 118 (13.45) | 42 (11.17) | 0.267 |
| Blood glucose values, Mean ± SD, mmol/L | 7.60 ± 2.95 | 7.64 ± 3.06 | 7.51 ± 2.67 | 0.495 |
| Platelet count, Mean ± SD, 10^9^/L | 199.12 ± 62.33 | 200.34 ± 62.82 | 196.30 ± 61.14 | 0.294 |
| International normalized ratio, Mean ± SD | 0.96 ± 0.10 | 0.97 ± 0.10 | 0.96 ± 0.09 | 0.316 |
| GCS score, Median (IQR) | 12.00 (9.00 - 14.00) | 12.00 (9.00 - 14.00) | 12.00 (9.00 - 14.00) | 0.791 |
| Systolic blood pressure, Mean ± SD, mm Hg | 168.49 ± 27.02 | 168.99 ± 27.21 | 167.31 ± 26.56 | 0.315 |
| Diastolic blood pressure, Mean ± SD, mm Hg | 97.33 ± 17.16 | 97.54 ± 17.10 | 96.83 ± 17.32 | 0.502 |
| Onset-to-first CT time, Median (IQR), h | 2.27 (1.40 - 3.50) | 2.25 (1.40 - 3.42) | 2.38 (1.39 - 3.73) | 0.299 |
| Baseline hematoma volume, Median (IQR), mL | 12.24 (6.68 - 22.99) | 12.60 (6.94 - 23.39) | 11.38 (6.38 - 21.48) | 0.279 |
| Baseline IVH volume, Median (IQR), mL | 0.00 (0.00 - 0.84) | 0.00 (0.00 - 1.11) | 0.00 (0.00 - 0.41) | 0.346 |
| Hematoma sites, lobar, n (%) | 248 (19.79) | 169 (19.27) | 79 (21.01) | 0.479 |
| Presence of IVH at baseline, n (%) | 394 (31.44) | 285 (32.50) | 109 (28.99) | 0.220 |
| Hypodensities, n (%) | 492 (39.27) | 352 (40.14) | 140 (37.23) | 0.335 |
| Blend sign, n (%) | 303 (24.18) | 211 (24.06) | 92 (24.47) | 0.877 |
| Island sign, n (%) | 307 (24.50) | 221 (25.20) | 86 (22.87) | 0.380 |
| Frequency of imaging markers | 0.31 (0.00 - 0.74) | 0.33 (0.00 - 0.75) | 0.26 (0.00 - 0.70) | 0.261 |
| **Outcomes** |  |  |  |  |
| mRS score, median (IQR) | 3.00 (2.00 - 5.00) | 3.00 (2.00 - 5.00) | 3.00 (2.00 - 5.00) | 0.294 |
| mRS score 3 - 6, n (%) | 713 (56.90) | 511 (58.27) | 202 (53.72) | 0.137 |
| mRS score 4 - 6, n (%) | 603 (48.12) | 428 (48.80) | 175 (46.54) | 0.463 |
| mRS score 6, n (%) | 144 (11.49) | 105 (11.97) | 39 (10.37) | 0.416 |
| Abbreviations: CT indicates computed tomography; GCS, Glasgow Coma Scale; ICH, intracerebral hemorrhage; IQR, interquartile range; IVH, intraventricular hemorrhage; mRS, modified Rankin Scale; and SD, standard deviation | | | | |

| **Table S2.** Cut-off values and predictive power for poor outcome (mRS score 3 - 6) of each continuous variable in the derivation cohort. | | | | | | |
| --- | --- | --- | --- | --- | --- | --- |
| **Variables** | **Cut**-**off** | **Sensitivity (95% CI)** | **Specificity (95% CI)** | **PPV (95% CI)** | **NPV (95% CI)** | **AUC (95% CI)** |
| Age, y | 68.50 | 0.37 (0.33 - 0.41) | 0.80 (0.76 - 0.84) | 0.72 (0.67 - 0.78) | 0.48 (0.44 - 0.52) | 0.61 (0.57 - 0.64) |
| Baseline hematoma volume, mL | 12.40 | 0.71 (0.66 - 0.75) | 0.66 (0.62 - 0.70) | 0.60 (0.55 - 0.64) | 0.76 (0.72 - 0.80) | 0.76 (0.73 - 0.79) |
| Baseline IVH volume, mL | 0.22 | 0.87 (0.83 - 0.90) | 0.42 (0.38 - 0.46) | 0.52 (0.48 - 0.56) | 0.81 (0.77 - 0.86) | 0.65 (0.62 - 0.68) |
| GCS score | 12.50 | 0.75 (0.71 - 0.79) | 0.70 (0.65 - 0.74) | 0.78 (0.74 - 0.81) | 0.67 (0.62 - 0.71) | 0.78 (0.75 - 0.81) |
| Platelet count, 10^9^/L | 178.50 | 0.28 (0.24 - 0.33) | 0.58 (0.53 - 0.62) | 0.32 (0.27 - 0.37) | 0.53 (0.49 - 0.57) | 0.58 (0.54 - 0.61) |
| Frequency of imaging markers | 0.27 | 0.68 (0.63 - 0.73) | 0.68 (0.64 - 0.72) | 0.60 (0.56 - 0.65) | 0.75 (0.71 - 0.79) | 0.72 (0.69 - 0.75) |
| Abbreviations: AUC indicates area under the curve; CI, confidence interval; GCS, Glasgow Coma Scale; IVH, intraventricular hemorrhage; NPV, negative predictive value; and PPV, positive predictive value. | | | | | | |

**Table S3. Calibration performance of the HAGIV score for predicting poor outcome (mRS score 3** - **6) in the derivation and validation cohorts**

| **Metric** | **Derivation cohort** | **Validation cohort** |
| --- | --- | --- |
| C-statistic | 0.863 | 0.836 |
| Brier score | 0.147 | 0.161 |
| Calibration intercept | 0.000 | -0.036 |
| Calibration slope | 1.000 | 0.803 |
| Emax | 0.007 | 0.051 |
| E90 | 0.007 | 0.051 |
| Eavg | 0.004 | 0.027 |
| Spiegelhalter z | 0.048 | 1.926 |
| Spiegelhalter P value | 0.962 | 0.054 |

Abbreviations: Emax indicates maximum absolute calibration error; E90, 90th percentile absolute calibration error; Eavg, average absolute calibration error.

| **Table S4.** Comparison of predictive power of the HAGIV score and other ICH scores for predicting poor outcome (mRS score 4 - 6) | | | | | | |
| --- | --- | --- | --- | --- | --- | --- |
| **ICH scores** | **Sensitivity (95% CI)** | **Specificity (95% CI)** | **PPV (95% CI)** | **NPV (95% CI)** | **AUC (95% CI)** | **DeLong's test** |
| **Derivation Cohort (*n* = 877)** |  |  |  |  |  |  |
| HAGIV score | 0.86 (0.82 - 0.89) | 0.70 (0.66 - 0.74) | 0.73 (0.69 - 0.77) | 0.84 (0.79 - 0.87) | 0.86 (0.84 - 0.88) | ... |
| ICH score | 0.58 (0.53 - 0.63) | 0.89 (0.86 - 0.92) | 0.84 (0.79 - 0.88) | 0.69 (0.65 - 0.73) | 0.79 (0.76 - 0.82) | <0.001 |
| MICH score | 0.62 (0.57 - 0.67) | 0.86 (0.82 - 0.89) | 0.81 (0.76 - 0.85) | 0.70 (0.66 - 0.74) | 0.80 (0.77 - 0.83) | <0.001 |
| Outcome score | 0.60 (0.55 - 0.65) | 0.75 (0.71 - 0.79) | 0.70 (0.65 - 0.74) | 0.66 (0.62 - 0.71) | 0.74 (0.71 - 0.77) | <0.001 |
| Landseed ICH score | 0.58 (0.53 - 0.63) | 0.87 (0.84 - 0.90) | 0.81 (0.76 - 0.85) | 0.68 (0.64 - 0.72) | 0.78 (0.75 - 0.81) | <0.001 |
| **Validation Cohort (*n* = 376)** |  |  |  |  |  |  |
| HAGIV score | 0.79 (0.73 - 0.85) | 0.71 (0.64 - 0.77) | 0.71 (0.64 - 0.77) | 0.80 (0.73 - 0.85) | 0.83 (0.79 - 0.87) | ... |
| ICH score | 0.53 (0.45 - 0.60) | 0.86 (0.80 - 0.91) | 0.77 (0.68 - 0.84) | 0.68 (0.61 - 0.73) | 0.76 (0.72 - 0.81) | 0.001 |
| MICH score | 0.59 (0.51 - 0.66) | 0.86 (0.80 - 0.90) | 0.78 (0.70 - 0.85) | 0.70 (0.64 - 0.76) | 0.77 (0.73 - 0.82) | 0.003 |
| Outcome score | 0.68 (0.61 - 0.75) | 0.73 (0.66 - 0.79) | 0.68 (0.61 - 0.75) | 0.72 (0.66 - 0.78) | 0.76 (0.71 - 0.81) | 0.006 |
| Landseed ICH score | 0.51 (0.43 - 0.58) | 0.85 (0.79 - 0.90) | 0.75 (0.66 - 0.82) | 0.67 (0.60 - 0.72) | 0.74 (0.69 - 0.78) | <0.001 |
| Abbreviations: AUC indicates area under the curve; CI, confidence interval; NPV, negative predictive value; ICH, intracerebral hemorrhage; and PPV, positive predictive value.  The HAGIV score consists of age, Glasgow Coma Scale score, baseline hematoma volume, frequency of imaging markers, and presence of intraventricular hemorrhage at baseline.  The ICH score consists of age, Glasgow Coma Scale score, baseline hematoma volume, and hematoma location.  The MICH score consists of Glasgow Coma Scale score, baseline hematoma volume, and presence of intraventricular hemorrhage at baseline.  The Outcome score consists of age, Glasgow Coma Scale score, and systolic blood pressure.  The Landseed ICH score consists of Glasgow Coma Scale score, baseline hematoma volume, and diabetes mellitus. | | | | | | |

| **Table S5.** Comparison of predictive power of the HAGIV score and other ICH scores for predicting poor outcome (mortality, mRS score 6) | | | | | | |
| --- | --- | --- | --- | --- | --- | --- |
| **ICH scores** | **Sensitivity (95% CI)** | **Specificity (95% CI)** | **PPV (95% CI)** | **NPV (95% CI)** | **AUC (95% CI)** | **DeLong's test** |
| **Derivation Cohort (*n* = 877)** |  |  |  |  |  |  |
| HAGIV score | 0.86 (0.78 - 0.92) | 0.68 (0.64 - 0.71) | 0.27 (0.22 - 0.32) | 0.97 (0.95 - 0.98) | 0.84 (0.80 - 0.87) | ... |
| ICH score | 0.80 (0.71 - 0.87) | 0.72 (0.69 - 0.76) | 0.28 (0.23 - 0.34) | 0.96 (0.95 - 0.98) | 0.82 (0.78 - 0.85) | 0.188 |
| MICH score | 0.83 (0.74 - 0.90) | 0.69 (0.65 - 0.72) | 0.26 (0.22 - 0.32) | 0.97 (0.95 - 0.98) | 0.81 (0.77 - 0.85) | 0.058 |
| Outcome score | 0.71 (0.62 - 0.80) | 0.76 (0.73 - 0.79) | 0.29 (0.24 - 0.35) | 0.95 (0.93 - 0.97) | 0.81 (0.77 - 0.85) | 0.226 |
| Landseed ICH score | 0.74 (0.65 - 0.82) | 0.70 (0.67 - 0.74) | 0.25 (0.21 - 0.31) | 0.95 (0.93 - 0.97) | 0.79 (0.75 - 0.83) | 0.006 |
| **Validation Cohort (*n* = 376)** |  |  |  |  |  |  |
| HAGIV score | 0.82 (0.66 - 0.92) | 0.67 (0.62 - 0.72) | 0.23 (0.16 - 0.30) | 0.97 (0.94 - 0.99) | 0.84 (0.78 - 0.90) | ... |
| ICH score | 0.79 (0.64 - 0.91) | 0.74 (0.69 - 0.78) | 0.26 (0.18 - 0.35) | 0.97 (0.94 - 0.99) | 0.81 (0.76 - 0.87) | 0.293 |
| MICH score | 0.85 (0.69 - 0.94) | 0.71 (0.65 - 0.75) | 0.25 (0.18 - 0.33) | 0.98 (0.95 - 0.99) | 0.83 (0.77 - 0.88) | 0.501 |
| Outcome score | 0.72 (0.55 - 0.85) | 0.72 (0.67 - 0.77) | 0.23 (0.16 - 0.31) | 0.96 (0.92 - 0.98) | 0.78 (0.71 - 0.85) | 0.114 |
| Landseed ICH score | 0.67 (0.50 - 0.81) | 0.72 (0.67 - 0.77) | 0.22 (0.15 - 0.30) | 0.95 (0.92 - 0.97) | 0.75 (0.68 - 0.82) | <0.001 |
| Abbreviations: AUC indicates area under the curve; CI, confidence interval; NPV, negative predictive value; ICH, intracerebral hemorrhage; and PPV, positive predictive value.  The HAGIV score consists of age, Glasgow Coma Scale score, baseline hematoma volume, frequency of imaging markers, and presence of intraventricular hemorrhage at baseline.  The ICH score consists of age, Glasgow Coma Scale score, baseline hematoma volume, and hematoma location.  The MICH score consists of Glasgow Coma Scale score, baseline hematoma volume, and presence of intraventricular hemorrhage at baseline.  The Outcome score consists of age, Glasgow Coma Scale score, and systolic blood pressure.  The Landseed ICH score consists of Glasgow Coma Scale score, baseline hematoma volume, and diabetes mellitus. | | | | | | |

| **Table S6. Predictive performance of the HAGIV score for poor outcome (mRS Score 3** - **6) stratified by baseline hematoma volume** | | | | | | | |
| --- | --- | --- | --- | --- | --- | --- | --- |
| **Cohort** | **Baseline hematoma volume** | **Poor outcome, *n* (%)** | **AUC (95% CI)** | **Sensitivity (95% CI)** | **Specificity (95% CI)** | **PPV (95% CI)** | **NPV (95% CI)** |
| Derivation | ≤30 mL | 375/730 (51.37%) | 0.84 (0.81 - 0.86) | 0.74 (0.69 - 0.78) | 0.77 (0.72 - 0.81) | 0.77 (0.72 - 0.81) | 0.73 (0.69 - 0.78) |
| Derivation | >30 mL | 136/147 (92.52%) | 0.86 (0.76 - 0.94) | 0.98 (0.94 - 0.99) | 0.27 (0.10 - 0.57) | 0.94 (0.89 - 0.97) | 0.50 (0.19 - 0.81) |
| Validation | ≤30 mL | 148/313 (47.28%) | 0.82 (0.78 - 0.87) | 0.72 (0.64 - 0.78) | 0.81 (0.74 - 0.86) | 0.77 (0.69 - 0.83) | 0.76 (0.69 - 0.82) |
| Validation | >30 mL | 54/63 (85.71%) | 0.62 (0.38 - 0.83) | 0.94 (0.85 - 0.98) | 0.11 (0.02 - 0.43) | 0.86 (0.75 - 0.93) | 0.25 (0.05 - 0.70) |

Abbreviations: AUC indicates area under the curve; CI, confidence interval; NPV, negative predictive value; and PPV, positive predictive value.

The HAGIV score consists of age, Glasgow Coma Scale score, baseline hematoma volume, frequency of imaging markers, and presence of intraventricular hemorrhage at baseline.


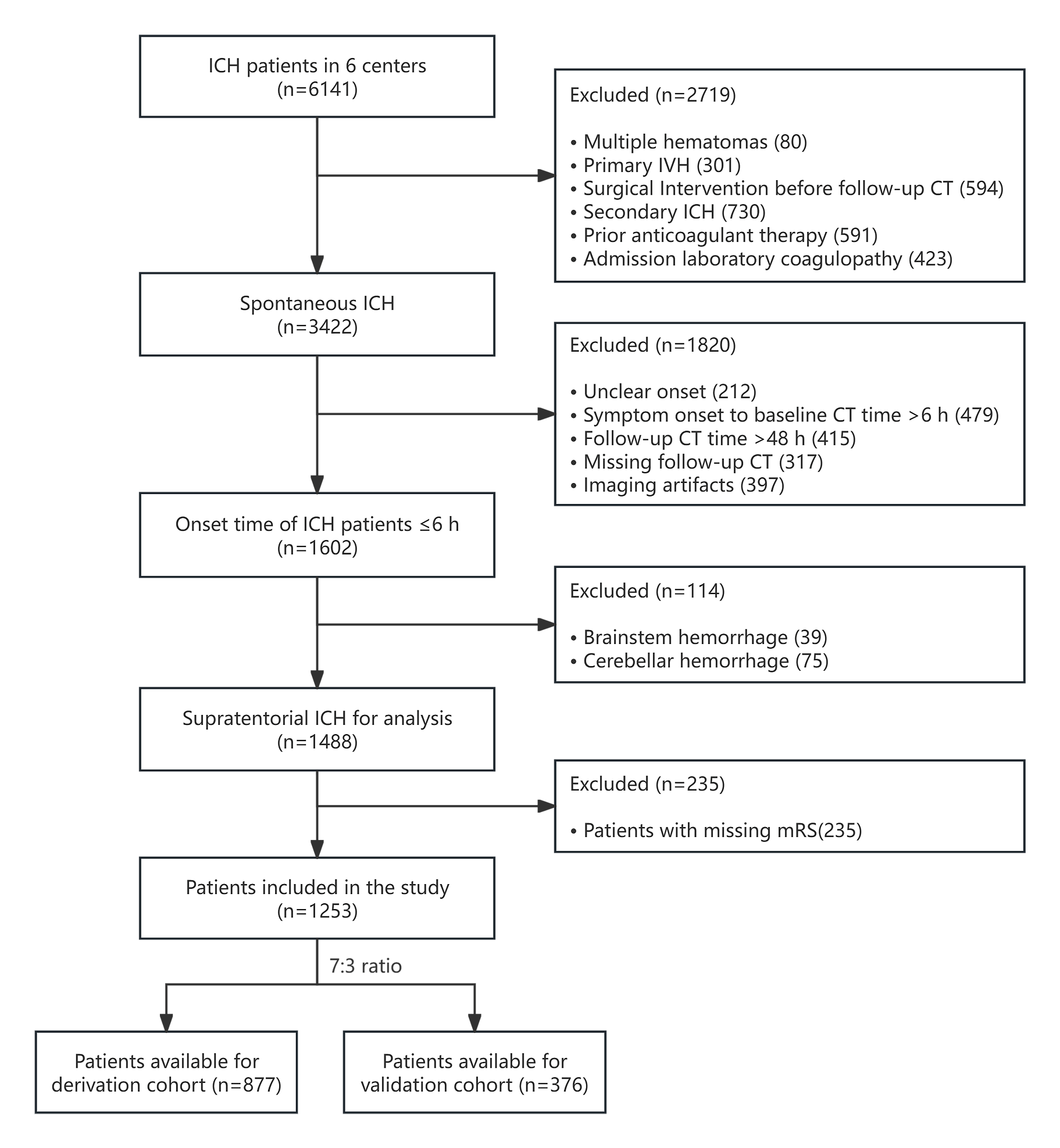


**Figure S1**. Study inclusion and exclusion flowchart.

Abbreviations: CT indicates computed tomography; ICH, intracerebral hemorrhage; and mRS, modified Rankin Scale.


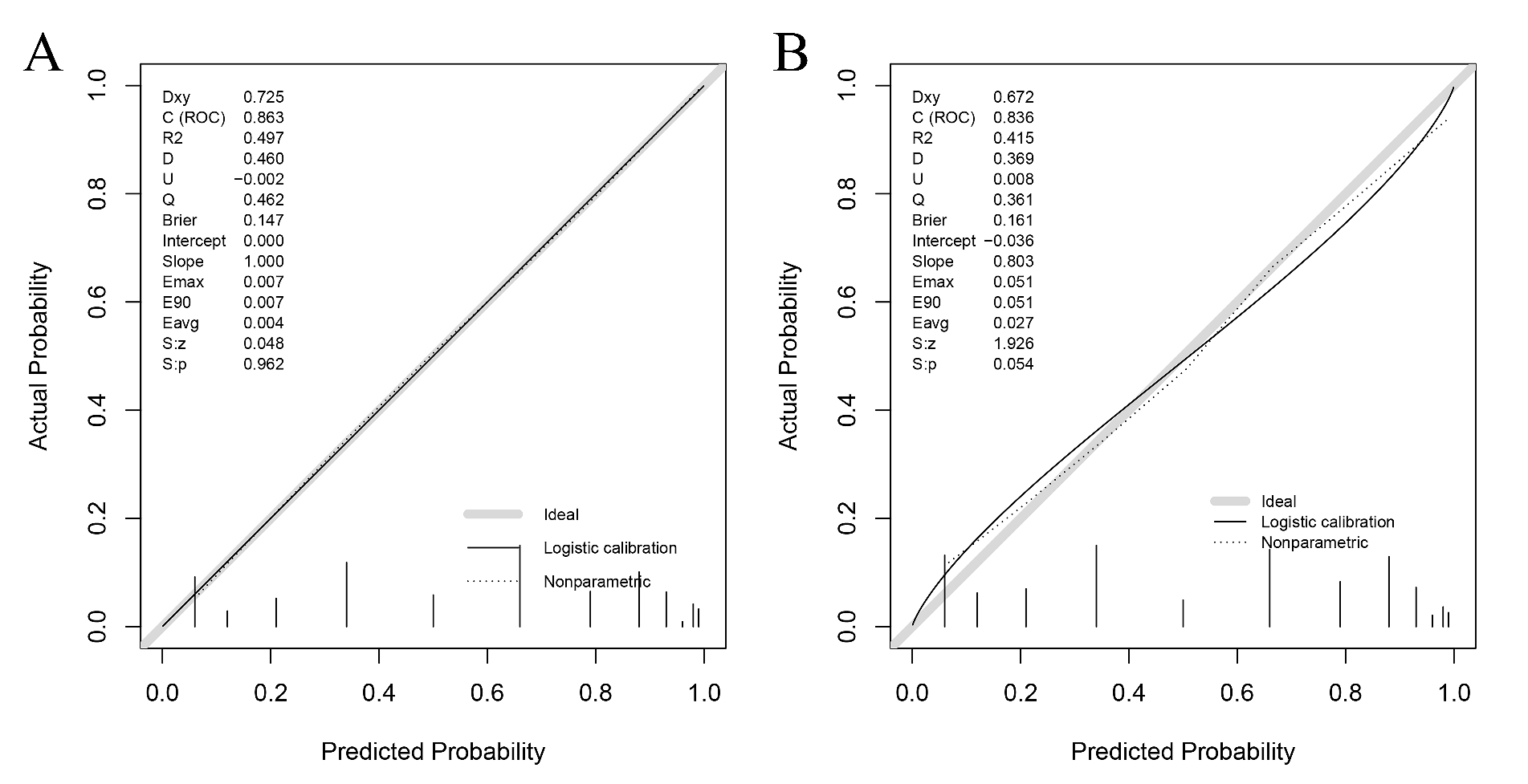


**Figure S2.** Calibration plots of the HAGIV score for predicting poor outcome (mRS score 3 - 6) in the derivation (A) and validation (B) cohorts.

Abbreviations: Emax indicates maximum absolute calibration error; E90, 90th percentile absolute calibration error; Eavg, average absolute calibration error; mRS, modified Rankin Scale; HAGIV, hematoma volume, age, Glasgow Coma Scale score, frequency of imaging markers, and intraventricular hemorrhage score


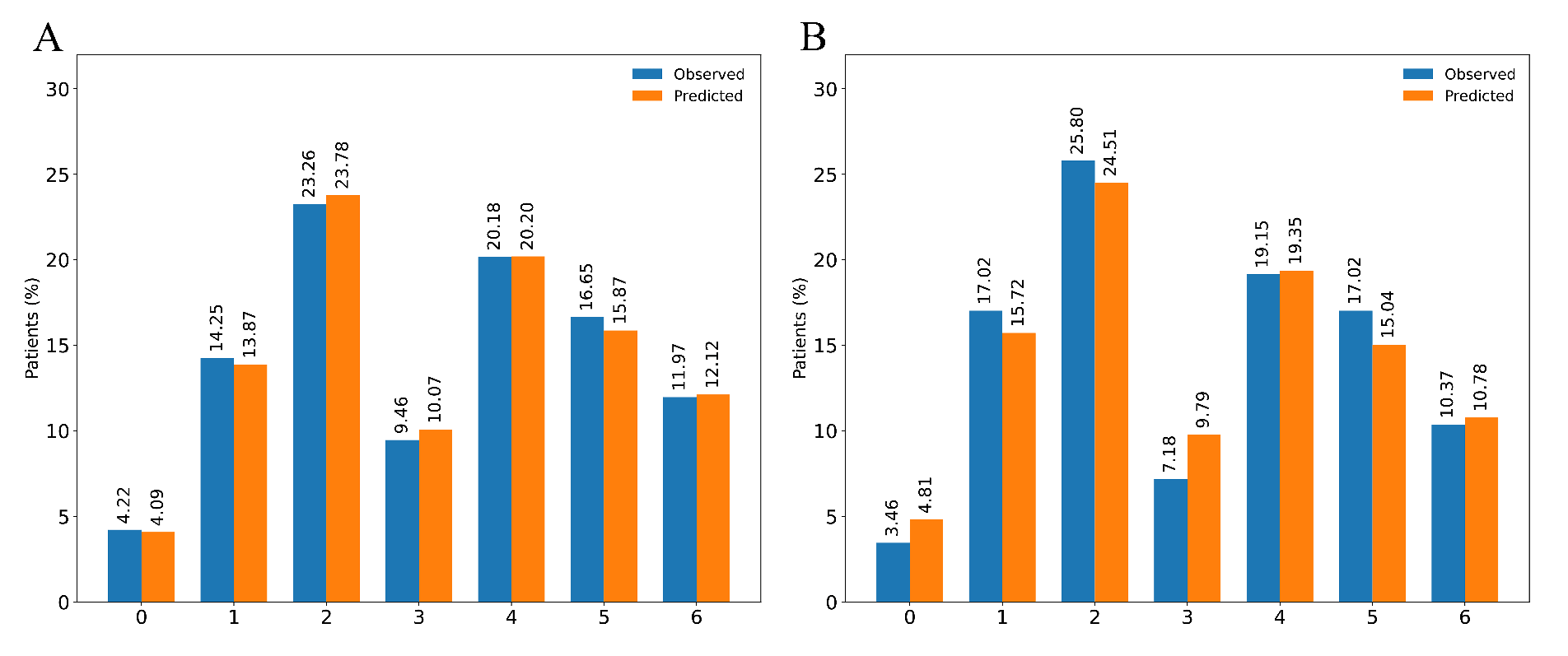


**Figure S3.** Observed and model-predicted 90-day modified Rankin Scale distributions in the derivation (A) and validation (B) cohorts.
